# I-SMEL a Big Catch!

**DOI:** 10.1021/acscentsci.3c01343

**Published:** 2023-11-08

**Authors:** Olivia Matsumoto-Elliott, Laura M. Sanchez

**Affiliations:** Department of Chemistry and Biochemistry, University of California, Santa Cruz, 1156 High St, Santa Cruz, California 95064, United States

Efficiently sampling the marine environment for new or known natural products, while maintaining the integrity of the ecosystem, is a notoriously difficult feat. In this issue of *ACS Central Science*, a team of researchers led by Charlotte Simmler offer a creative solution through the creation of the ‘In Situ Marine moleculELogger’ aka the I-SMEL device.^1^

The ocean is a harsh environment that harbors a high amount of biodiversity which in turn fosters the biosynthesis of complex, natural products with unique chemical scaffolds. The ocean covers 70% of the Earth, and we have yet to explore the majority of this area. We have only been able to access marine organisms at depth with the patenting of “self-contained underwater breathing apparatus” (SCUBA) and the invention of submersible technologies. Exploration of the marine environment by SCUBA has led to the discovery of a number of exciting marine natural products which have advanced to clinical use and drug trials including salinosporamide A (NPI-0052, Marizomib), a derivative of dolastatin 10 (brentuximab vedotin), and halichondrin B (Halaven), among others.^2^ Additionally, chemical ecology studies have expanded our understanding of how toxins form, accumulate, and impact animals in the marine environment.^3^

Many researchers, both historically and presently, directly sample the rich marine environment by removing marine organisms and preserving or extracting them in the field. However, a number of factors have facilitated a re-examination of how we might access these precious organisms, namely preserving the rights from the country in which these organisms were sampled^4^ and maintaining the integrity of the marine environment, which is an incredibly limited resource that is evolving due to the changing climate.^5^ As an extreme example, halichondrin B’s structure was famously elucidated after 600 kg (nearly 1 ton) of sponge was collected off the coast of the Miura Peninsula in Japan over a 6-month period.^6^ This type of sampling campaign is simply not feasible or sustainable today.

Notable attempts have been made to sample the marine environment with the aim of preserving marine organisms **(**Figure 1**)**. Early attempts at isolating these compounds from seawater included solvent extraction, absorption into activated charcoal, or using ion exchange resins.^7^ Although reverse-phase liquid chromatography (LC) with C18 columns yielded promising results, Coll et al. recognized that water-borne compounds required enrichment that could be addressed by passing large volumes of seawater sampled within the vicinity of the excreting organism, and thus, made one of the earliest *in situ* submersible sampling devices.^7^ In their design, they made a chamber to encompass the organism of interest while a pump connected to a manifold pushed water simultaneously through four C18 cartridges. Sampling and analysis methods were limited, and popularity of this device was thus limited. We also speculate that the size and weight of the device would not be easily compatible for use by a single SCUBA diver.

**Figure 1:**
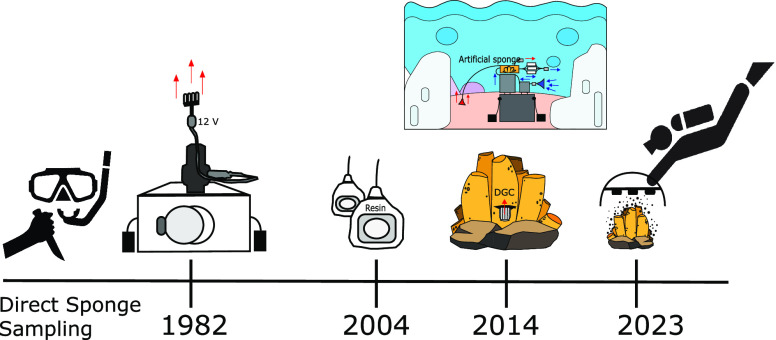
Timeline of sampling the marine molecular environment. Early sampling included direct excision of sponge tissue by divers. In **1982**, the first submersible sampling apparatus was deployed.^7^ Over 20 years later, in **2004**, solid phase adsorption toxin tracking (SPATT) resin bags were employed.^8^ Ten years later, in **2014**, a submersible artificial sponge apparatus^9^ and diffusion growth chamber (DGC) were described.^10^ Now finally in **2023**, the In Situ Marine moleculE Logger (I-SMEL) has been introduced to maintain the integrity of the marine environment while sampling.^1^

Many natural products are present only in very low concentrations in the sponge itself; lab cultivation of the producing organism would facilitate a more direct route to study and scale production of desired natural products. However, sponges and their symbiotic bacteria have proven difficult to cultivate *in vitro*, with only 0.06–11% of the sponge-associated bacterial community being cultivated as of 2007.^10^ To overcome the historical challenges with sponge aquaculture, Steinert et al. proposed using a diffusion growth chamber (DGC) method, where bacteria could be cultivated *in situ*.^10^ Microtube filters were placed in chambers which allowed nutrients from the sponge microenvironment to freely diffuse. Insertion of the DGCs into the sponge was successful for some sponges that could accommodate the chamber size, but other sponges either could not fit the chamber size or rejected the chamber, resulting in hole formation as the sponge tissue retracted.

Mechanical replication of a natural sponge to initiate colonization by native sponge microbes has also been attempted in order to accumulate natural products, leaving native sponges undisturbed.^9^ A pump system was employed with resins to concentrate compounds that flowed through the artificial sponge. Unfortunately, the origin of the detected natural products (sponge vs microbe) was inconclusive, and production by adjacent sponges could not be ruled out. Other approaches involved simply deploying resins in contained vessels to sample ocean environments. Solid phase adsorption toxin tracking (SPATT) employs resin-filled sachets but can only detect extracellular biotoxins and lacks calibration and validation techniques in addition to being limited in collection capacity.^8^

Despite great interest and biomedically relevant application of marine natural products, inconsistent access to producing organisms and difficulty recapitulating the sponge environment in the laboratory setting continue to make this a challenging field of study. *In vitro* studies have been thwarted by lab cultivation-resistant bacteria, not allowing for scalability of compounds of interest. Just this year, Hesp et al. was able to create the first continuous marine sponge cell line originating from *Geodia barretti* for industrial scalable sponge cells.^11^ The in situ methods described above have some level of difficulty in the acquisition of compounds in different seascapes, at different depths, and difficulty with identifying the producing organism.

Taking these previous sampling strategies into account, the I-SMEL device is highly innovative and exciting because I-SMEL does not require the researchers to remove the organisms from their native habitat. This new device, whose design is well described in a recent *ACS Central Science* paper,^1^ can be handled by a single SCUBA diver and was field tested in three unique experiments to demonstrate its utility (Figure 2). In Experiment 1, which focused on underwater sponge communities, 10 L of seawater (2 L filtered at five sampling sites) was passed through three different SPE cartridges and examined using LC-MS/MS. This facilitated the description of the “average chemical seascape” and highlighted that this device could indeed capture compound classes including alkaloids, fatty acids, terpenoids, polyketides, and peptidic compounds. Experiment 2 sought to profile exometabolites from specific sponge species sampled from five different individual sponges, while Experiment 3 tested intraindividual variability by sampling the same sponge three different times. For these experiments, the analytes obtained from the I-SMEL device were compared to extracted pieces of the sponge itself. A major highlight of this manuscript was the rigorous characterization of the exometabolites by the team and the detailed spectral interpretation which supported their conclusions; the I-SMEL is a nondestructive approach to collect a standardized volume of seawater to capture and enrich, in a temporally and spatially informed manner, exometabolites released by marine organisms.

**Figure 2:**
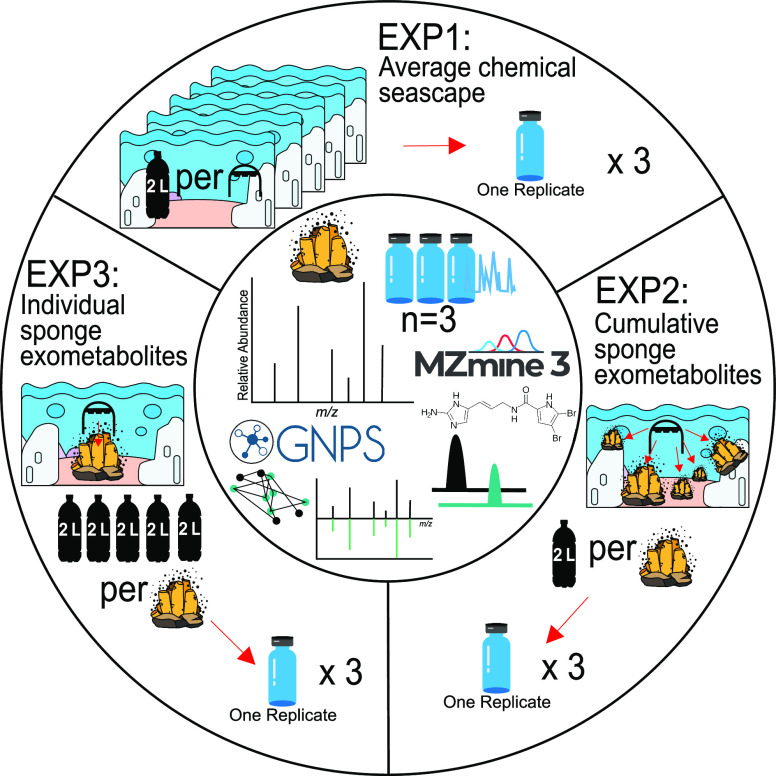
Summary of EXP1, EXP2, and EXP3. The I-SMEL was evaluated using three different collection experiments. Experiment 1 - the average seascape was studied. Experiment 2 - the cumulative sponge exometabolites were captured, with five different individual sponges examined across three species. Experiment 3 - an individual sponge’s exometabolites collected at three different time points were measured.

The future research potential of the I-SMEL device is likely to be expansive. I-SMEL offers a stringent, robust way to study marine organisms and the compounds they produce in their native environment with minimal disturbance to the organisms and their surrounding environments. This could facilitate the study of endangered species in marine protected areas, seasonal variations for exometabolite production, and monitoring of toxins or human impacts in the marine environment, and could uncover new sites or habitats that have high levels of chemodiversity prior to removing organisms from the marine environment. The possibilities are endless. This is a truly creative and rigorous approach toward discovering new marine natural products and facilitating chemical ecology studies.

## References

[ref1] M., Mauduit; Derrien M.; Grenier M.; Greff S.; Molinari S.; Chevaldonné P. Charlotte Simmler, Thierry Pérez. In Situ Capture and Real Time Enrichment of Marine Chemical Diversity. ACS Central Science. 2023 9 10.1021/acscentsci.3c00661 PMC10683479 38033807

[ref2] Jiménez C. Marine Natural Products in Medicinal Chemistry. ACS Med. Chem. Lett. 2018, 9 (10), 959–961. 10.1021/acsmedchemlett.8b00368. 30344898 PMC6187399

[ref3] Selander E.; Kubanek J.; Hamberg M.; Andersson M. X.; Cervin G.; Pavia H. Predator Lipids Induce Paralytic Shellfish Toxins in Bloom-Forming Algae. Proc. Natl. Acad. Sci. U. S. A. 2015, 112 (20), 6395–6400. 10.1073/pnas.1420154112. 25918403 PMC4443330

[ref4] Nagoya Protocol on Access to Genetic Resources and the Fair and Equitable Sharing of Benefits Arising from Their Utilization to the Convention on Biological Diversity: Text and Annex; Secretariat of the Convention on Biological Diversity, United Nations Environmental Programme, 2010.

[ref5] Sigwart J. D.; Blasiak R.; Jaspars M.; Jouffray J.-B.; Tasdemir D. Unlocking the Potential of Marine Biodiscovery. Nat. Prod. Rep. 2021, 38 (7), 1235–1242. 10.1039/D0NP00067A. 34287433

[ref6] Hirata Y.; Uemura D. Halichondrins - Antitumor Polyether Macrolides from a Marine Sponge. J. Macromol. Sci. Part A Pure Appl. Chem. 1986, 58 (5), 701–710. 10.1351/pac198658050701.

[ref7] Coll J. C.; Bowden B. F.; Tapiolas D. M.; Dunlap W. C. In Situ Isolation of Allelochemicals Released from Soft Corals (Coelenterata: Octocorallia): A Totally Submersible Sampling Apparatus. J. Exp. Mar. Bio. Ecol. 1982, 60 (2), 293–299. 10.1016/0022-0981(82)90166-6.

[ref8] MacKenzie L.; Beuzenberg V.; Holland P.; McNabb P.; Selwood A. Solid Phase Adsorption Toxin Tracking (SPATT): A New Monitoring Tool That Simulates the Biotoxin Contamination of Filter Feeding Bivalves. Toxicon 2004, 44 (8), 901–918. 10.1016/j.toxicon.2004.08.020. 15530973

[ref9] La Clair J. J.; Loveridge S. T.; Tenney K.; O’Neil-Johnson M.; Chapman E.; Crews P. In Situ Natural Product Discovery via an Artificial Marine Sponge. PLoS One 2014, 9 (7), e100474 10.1371/journal.pone.0100474. 25004127 PMC4086721

[ref10] Steinert G.; Whitfield S.; Taylor M. W.; Thoms C.; Schupp P. J. Application of Diffusion Growth Chambers for the Cultivation of Marine Sponge-Associated Bacteria. Mar. Biotechnol. 2014, 16 (5), 594–603. 10.1007/s10126-014-9575-y. 24838766

[ref11] Hesp K.; van der Heijden J. M. E.; Munroe S.; Sipkema D.; Martens D. E.; Wijffels R. H.; Pomponi S. A. First Continuous Marine Sponge Cell Line Established. Sci. Rep. 2023, 13 (1), 5766 10.1038/s41598-023-32394-x. 37031251 PMC10082835

